# Three-dimensional carbon coated and high mass-loaded NiO@Ni foam anode with high specific capacity for lithium ion batteries†

**DOI:** 10.1039/d4ra07119k

**Published:** 2024-12-23

**Authors:** Nurbolat Issatayev, Diana Abdumutaliyeva, Yerbolat Tashenov, Dossym Yeskozha, Adilkhan Seipiyev, Zhumabay Bakenov, Arailym Nurpeissova

**Affiliations:** a Institute of Batteries 53 Kabanbay Batyr Ave. Astana 010000 Kazakhstan; b Department of Chemistry, L. N. Gumilyov Eurasian National University 2 Satpayev St. Astana 010008 Kazakhstan; c National Laboratory Astana, Nazarbayev University 53 Kabanbay Batyr Ave. Astana 010000 Kazakhstan arailym.nurpeissova@nu.edu.kz; d Department of Chemical and Materials Engineering, Nazarbayev University 53 Kabanbay Batyr Ave. Astana 010000 Kazakhstan

## Abstract

Nickel oxide (NiO) is known for its remarkable theoretical specific capacity, making it a highly appealing option for electrode materials in electrochemical energy storage applications. Nevertheless, its practical use is limited by poor electrochemical performance and complicated electrode fabrication processes. To address these issues, we propose a new anode design comprising an intermediate NiO nanoarray layer and a carbon coating layer grown directly on a three-dimensional (3D) conductive nickel foam substrate, designated as C@NiO@Ni foam. This anode with a high NiO mass loading of 5–6 mg cm^−2^ is fabricated by a two-step process: thermal oxidation of the nickel foam, followed by carbon coating. The 3D architecture, with its large surface area, significantly enhances the contact between the electrode and electrolyte, thereby shortening the Li-ion diffusion pathway. Additionally, the carbon layer plays a crucial role in accommodating the volume changes of NiO during cycling, preventing the detachment of NiO from the Ni foam substrate, and enhancing the electronic conductivity of the C@NiO@Ni foam. The resulting porous C@NiO@Ni anode was thoroughly analyzed using scanning electron microscopy (SEM), X-ray diffraction (XRD), and energy-dispersive X-ray spectroscopy (EDS). When used as an anode material for lithium-ion batteries (LIBs), this anode showcased an impressive reversible capacity of around 678 mA h g^−1^ at 0.1C after 100 cycles. Furthermore, it demonstrated excellent electrochemical performance at a high current, sustaining a specific capacity of 387 mA h g^−1^ at 1C after 100 cycles.

## Abstract 2

Design and synthesis of carbon-coated NiO@Ni foam as a high-performance anode for next-generation lithium-ion batteries.

## Graphical abstract

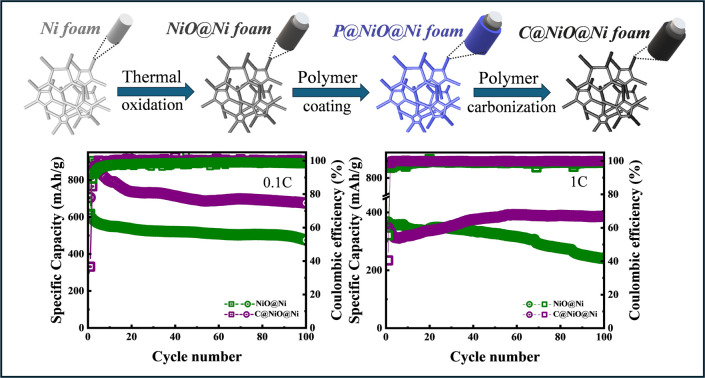

## Introduction

The graphite anode has succeeded remarkably in commercial lithium-ion batteries (LIBs). However, it faces an inherent capacity limit of 372 mA h g^−1^, which poses challenges for developing next-generation energy storage systems.^1,2^ This limitation has generated strong interest in finding electrode materials with higher specific capacity. Therefore, considerable research is directed toward developing new anode materials that not only provide higher reversible specific capacity but also improve cycling stability.^3^

Transition metal oxides (TMOs) are promising candidates for anode materials due to their high theoretical capacities, abundant availability, and cost-effectiveness.^4^ Notably, NiO stands out with a theoretical capacity of 718 mA h g^−1^, making it particularly attractive because of its affordability, low toxicity, and superior safety compared to other TMOs.^5^ Despite its potential, NiO faces several significant challenges that hinder its commercialization. These include low electrical conductivity, slow Li-ion storage kinetics, and rapid volume changes during the charging and discharging processes, contributing to rapid capacity decay.^6^ Additionally, the traditional manufacturing process for NiO is complex and involves adding conductive agents and binders, which reduces the overall energy density. Each coin cell usually contains only approximately 0.54–0.65 mg cm^−2^ of active material.^7,8^ Consequently, increasing both the mass loading and the specific capacity of NiO while minimizing its volume expansion remains a major challenge.

Recent research indicates that NiO nanostructures, such as nanoparticles, nanorods, and composite designs, can greatly improve performance by expanding the specific surface area of NiO and reducing the Li-ion diffusion distance.^9–11^ To further boost these improvements, constructing self-standing and binder-free three-dimensional (3D) electrodes has proven to be a particularly effective strategy.^12,13^ The self-supporting structure of 3D electrodes offers several key advantages, including a distinctive 3D design that provides numerous growth sites, allowing for higher loading and specific capacity without the need for additional conductive agents or binders.^14,15^ Moreover, the porous structure of the 3D conductive framework facilitates electrolyte penetration, speeds up Li-ion diffusion, and enhances interfacial reaction kinetics.^16^ For example, Y. Song *et al.* fabricated a 3D NiO@Ni foam electrode by thermal oxidation at 700 °C for 5 minutes. The electrode showed a reversible capacity of 550 mA h g^−1^ after 50 cycles at a current of 0.1 C.^17^ R. Song and co-workers synthesized metallic nickel aerogel using a straightforward hydrothermal reduction method. The aerogel was then annealed to obtain 3D porous NiO@Ni electrodes. The resulting 3D NiO/Ni anode exhibited a high areal capacity of 1.93 mA h cm^−1^ at a current density of 1 mA cm^−2^, along with good cycling stability.^18^

Another effective strategy for mitigating the volume expansion of active materials is surface coating. This approach can be applied to both powder materials and electrode materials with a certain morphology. However, some coating materials may exhibit low ionic or electronic conductivity, which can lead to a decrease in the rate performance of the active materials.^19^ Carbon is an ideal coating material for electrodes, known for its excellent electrical conductivity, good environmental resistance, and low cost.^20^ For instance, J. Zhang and colleagues synthesized a biochar-carbon nanotube (CNT)-NiO composite and used it as an anode for LIBs which illustrates an excellent reversible capacity of 674.6 mA h g^−1^ after 100 cycles at a current density of 0.1C.^21^ In light of these considerations, the fabrication of carbon-coated nanoarray NiO anodes that could be grown directly on 3D nickel foam is both desirable and challenging.

Therefore, herein, we propose a porous three-dimensional C@NiO@Ni anode with a high mass loading of 5–6 mg cm^−2^, fabricated through thermal oxidation followed by carbon coating. The *in situ* growth of NiO on the surface of the 3D Ni foam enhances the adhesion between the active materials and the nickel substrate, allowing for high loading and reversible areal capacity without requiring conductive agents or binders. The abundant macropores and cross-linked network of 3D NiO@Ni foam establish a rapid and efficient transport framework for electrons and ions, thereby improving redox reaction kinetics. Moreover, the carbon coating effectively serves a dual purpose: it significantly mitigates the volume expansion of NiO that occurs during charge and discharge cycles, and it also enhances the electrical conductivity of the electrode. Consequently, when used as an anode material for LIBs, the prepared electrode showcased an impressive reversible capacity of around 678 mA h g^−1^ at 0.1C after 100 cycles. Furthermore, it exhibited excellent electrochemical performance at a high current, maintaining a specific capacity of 387 mA h g^−1^ at 1C after 100 cycles.

## Experimental part

### Materials

Ni foam (0.9 mm thickness, 0.62 g cm^−3^ bulk density, 93% porosity), poly(methyl methacrylate) (PMMA, average *M*_w_ = 100 000), polyacrylonitrile (PAN, average *M*_w_ = 150 000), and anhydrous *N*,*N*-dimethylformamide (DMF) were purchase from Sigma Aldrich (St. Louis, MO, Germany). All reagents were of analytical grade and used without additional purification.

### Synthesis of NiO@Ni foam

**Fig. 1:**
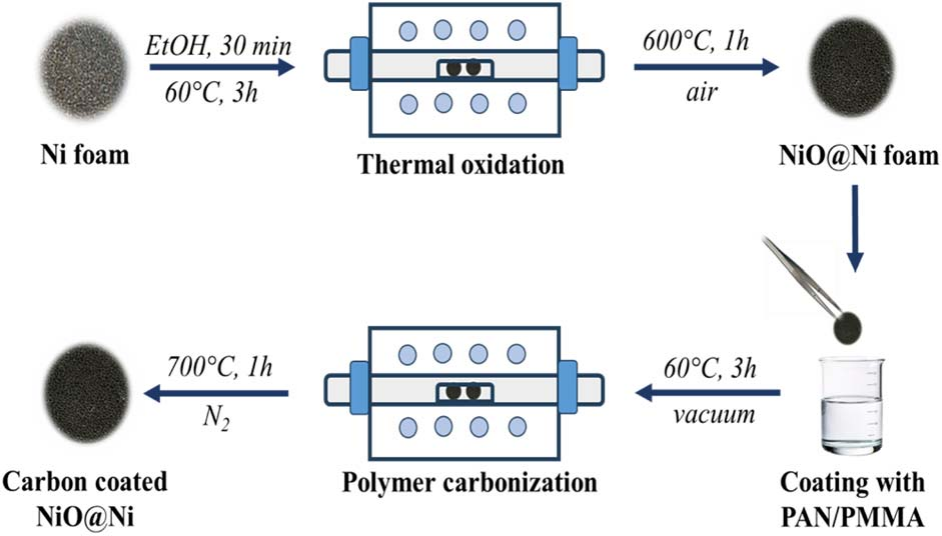
Schematic diagram of the synthesis route of C@NiO@Ni foam.

The porous NiO electrodes were fabricated by thermal oxidation of Ni foam in air as shown in Fig. 1. Initially, the Ni foam was cut into disks with 14 mm diameter. These disks were then thoroughly cleaned by ultrasonication in ethanol for 30 min to remove dust and dirt. After the cleaning process, the electrode precursors were dried in a vacuum oven at 60 °C for 3 h to ensure complete removal of residual moisture. The Ni foams were then placed in a tube furnace and heated at 600 °C for 1 h at a controlled rate of 5 °C per minute, resulting in the formation of NiO@Ni foams. The mass loadings of NiO were approximately 5–6 mg cm^−2^.

### Synthesis of C@NiO@Ni foam

The carbon coating of NiO@Ni foam was achieved by dip-coating the NiO@Ni foam into a 3% solution of PAN/PMMA (in a 1 : 1 mass ratio). After each dipping, a polymer-coated substrate was dried at 60 °C in a vacuum oven for 3 hours. This dip-coating and drying process was repeated several times to ensure a uniform polymer coating. Subsequently, the coated material underwent a pre-oxidation step in a muffle furnace at 280 °C for 1 hour to stabilize the coating. Finally, the stabilized material was annealed at 700 °C in a nitrogen atmosphere, with a ramping rate of 5 °C per minute. To study the effect of carbon coating on the electrochemical performance of NiO@Ni foam anode, the polymer coating on the surface of NiO@Ni foam was applied in 2, 4, and 6 layers, and the resulting samples were denoted as C@NiO@Ni foam/2, C@NiO@Ni foam/4, and C@NiO@Ni foam/6, respectively.

### Characterization

The structural analysis of fabricated materials was characterized by X-ray diffraction (XRD) using a Bruker D8 diffractometer with Cu Kα radiation (*λ* = 1.5418 Å), covering diffraction angles (2*θ*) from 10° to 90° at a scanning rate of 5° per minute. Scanning electron microscopy (SEM, Zeiss Crossbeam 540, Germany) coupled with energy-dispersive X-ray spectroscopy (EDS) was used to examine the film morphologies. Raman spectroscopy (Horiba LabRam Evolution) with a 532 nm laser was used to probe the spectral region between 1100 and 1700 cm^−1^.

### Electrochemical measurements

The electrochemical properties of the sample were assessed using CR2032 coin-type batteries assembled in an argon-filled glove box (MBRAUN, LABmaster Pro Glovebox, Germany) containing <0.1 ppm O_2_ and <0.1 ppm H_2_O. Lithium chips served as both the counter and reference electrode. The lithium hexafluorophosphate (LiPF_6_) dissolved in a mixture of ethylene carbonate (EC), diethyl carbonate (DEC), and ethyl methyl carbonate (EMC) in a volume ratio of 1 : 1 : 1 was utilized as an electrolyte. Celgard 2400, microporous polypropylene, was employed as the separator membrane. After assembly, the coin cells were allowed to rest overnight before undergoing testing with a Neware BTS4000 test (Neware Co., Shenzhen, China) system across a potential range of 0.01 to 3.0 V *vs.* Li/Li^+^. Cyclic voltammetry (CV) and electrochemical impedance spectroscopy (EIS) measurements were conducted using a BioLogic VMP3 Multichannel Potentiostat/Galvanostat (BioLogic Instruments, France). The mass of NiO in the NiO@Ni foam was used to calculate the current density, and the theoretical capacity was set at 718 mA h g^−1^. While the eqn (1) was used to calculate the theoretical capacity of the C@NiO@Ni foam:

1 *C*_composite_ = *w*_NiO_ × C_NiO_ + *w*_carbon_ × *C*_carbon_

where: *C*_NiO_ = 718 mA h g^−1^ and *C*_carbon_ = 200 mA h g^−1^.

## Result and discussion

C@NiO@Ni foam was prepared through a straightforward process involving the thermal oxidation of Ni foam, followed by polymer coating and subsequent carbonization in an inert atmosphere, as illustrated in Fig. 1. A PAN-PMMA solution was employed to create a carbon layer with a high specific surface area and an open-pore structure. During the carbonization process, PAN acted as the carbon precursor, forming a carbon layer on the surface of NiO@Ni foam. At the same time, PMMA functioned as a pore-forming agent, decomposing almost entirely at high temperatures to generate the pores. Due to the immiscibility of PAN and PMMA, the complete breakdown of PMMA resulted in uniformly distributed pores within the carbon layer.^22,23^

**Fig. 2:**
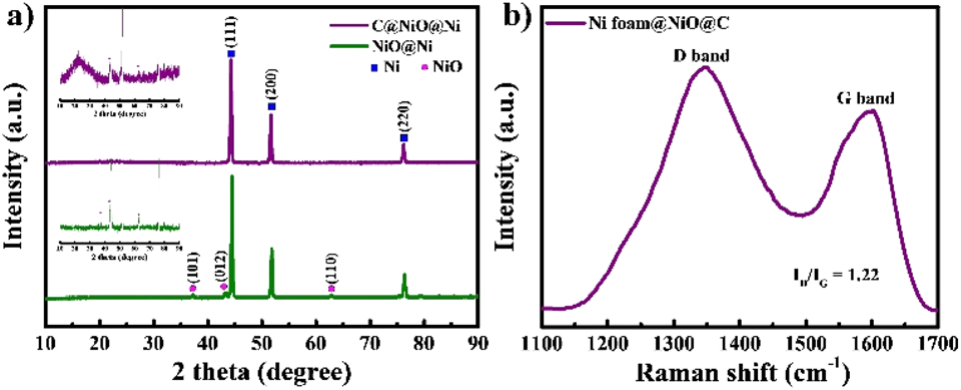
(a) XRD of NiO@Ni foam and C@NiO@Ni foam and (b) Raman of C@NiO@Ni foam.

The formation of NiO on the surface of Ni foam and its coating with carbon were studied by XRD (Fig. 2a). In the XRD pattern, three prominent diffraction peaks are observed at 44.5°, 51.8°, and 76.4°, corresponding to the (111), (200), and (220) planes of Ni, respectively.^24^ For both NiO@Ni foam and C@NiO@Ni, additional peaks observed at 37.4°, 43.4°, and 62.9° are attributed to the (101), (012), and (110) planes of NiO.^18^ A broad, extra peak at 23.6°, associated with the (002) graphitic plane, can be detected for C@NiO@Ni.^25^ Raman spectroscopy analysis was carried out to confirm further carbon formation on the surface of NiO@Ni foam (Fig. 2b). The Raman spectra revealed two distinct peaks: the G band at about 1585 cm^−1^, associated with carbon atoms in the sp^2^ electron configuration of graphite sheets, and the D band at about 1347 cm^−1^, indicating a disordered and defective structure of the carbon materials.^26^ The extent of disorder in the carbon material can be determined by the ratio of the integrated intensities of the D band to the G band (*I*_D_/*I*_G_), with higher values reflecting a greater number of defects in the carbon atoms.^25^ The *I*_D_/*I*_G_ ratio is 1.22 for C@NiO@Ni foam.

**Fig. 3:**
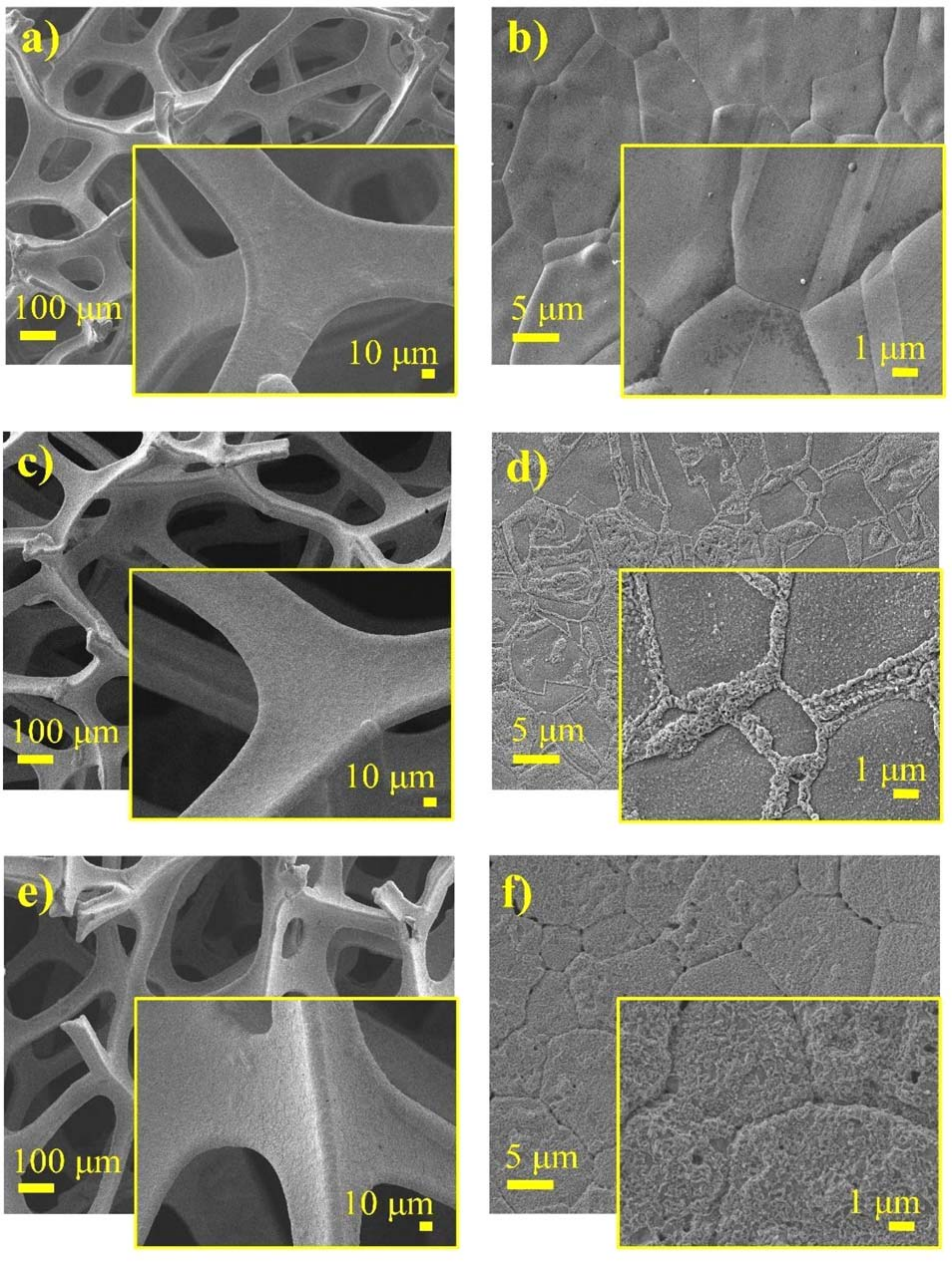
SEM images of (a and b) Ni foam, (c and d) NiO@Ni foam, and (e and f) C@NiO@Ni foam.

**Fig. 4:**
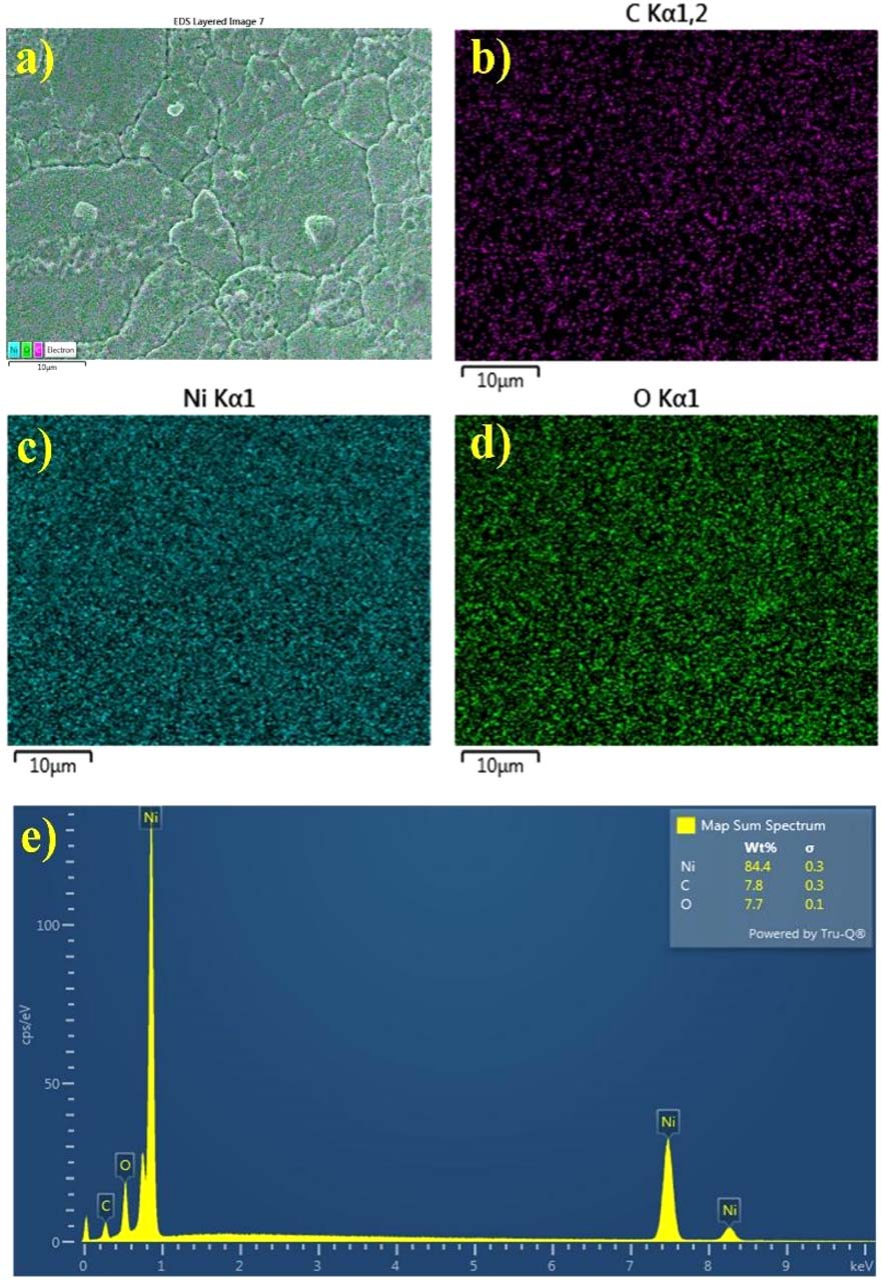
EDS mapping of C, Ni, and O elements on the (a–d) surface of C@NiO@Ni foam nanostructured branches and (e) EDS spectra.

The morphology and elemental analysis of pristine Ni foam, Ni foam coated with NiO, and NiO with carbon were analyzed using a scanning electron microscope (SEM) and an energy-dispersive X-ray spectroscopy (EDS) mapping, respectively, as illustrated in Fig. 3 and 4. SEM images of the pristine Ni foam revealed a highly porous and interconnected network of nickel filaments. The foam has a well-defined structure with uniform open pores throughout the surface, providing a large surface area. High-magnification images of nickel foam show a relatively smooth surface with clearly visible grain boundaries of metallic nickel. SEM images of pure Ni foam show a highly porous and interconnected network of nickel filaments (Fig. 3a and b). The foam has a well-defined structure with open pores uniformly distributed over its surface, contributing to its large surface area. High-magnification SEM images further highlight the relatively smooth surface of the nickel foam with distinct grain boundaries of metallic nickel. SEM images of NiO-coated and carbon–NiO-coated Ni foam indicate that the 3D structure of the Ni foam is well-preserved as shown in Fig. 3c–f. The surface displays increased roughness resulting from the growth of NiO and the incorporation of the carbon coating, highlighting the successful deposition of both NiO and carbon–NiO materials.

EDS mapping clearly reveals the uniform formation of the NiO layer along with its carbon coating. The distribution of oxygen and carbon elements in the Ni foam framework is consistent and well-defined (Fig. 4).

**Fig. 5:**
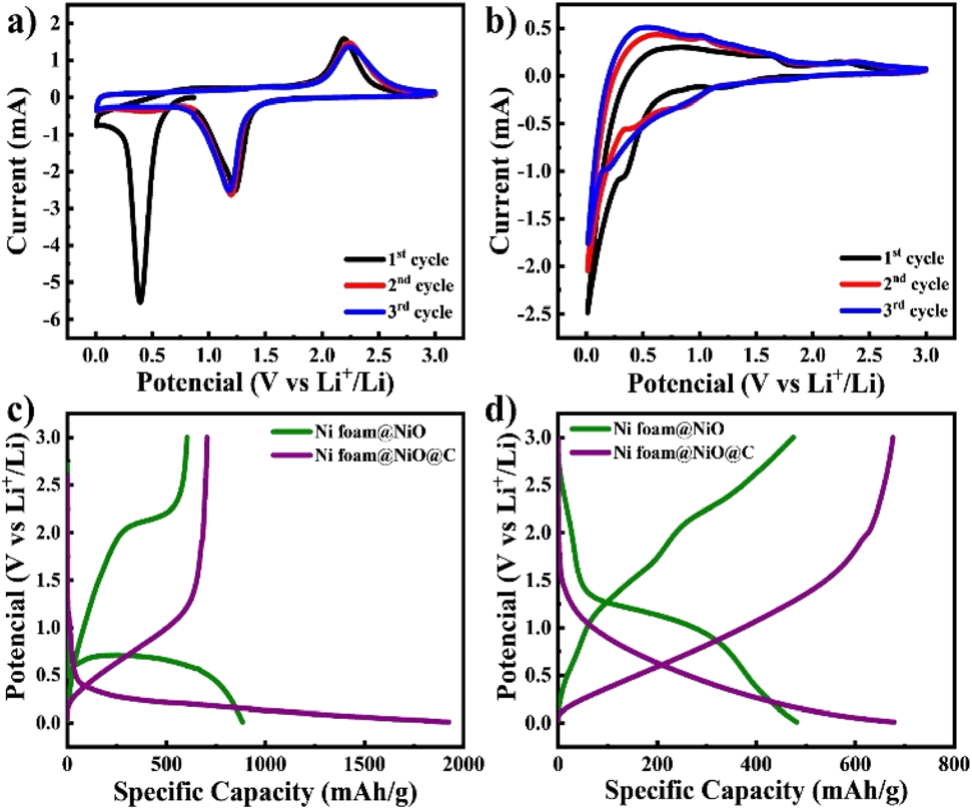
CV curves of (a) NiO@Ni foam and (b) C@NiO@Ni foam and galvanostatic charge–discharge curves of both electrodes at (c) the 1st cycle and (d) the 100th cycle at current density of 0.1C.

The fabricated electrodes were directly utilized as anodes for LIBs, followed by extensive electrochemical testing. CV curves were measured within a potential range of 0.01–3 V at a scan rate of 0.1 mV s^−1^ as illustrated in Fig. 5a and b. The initial CV curve of NiO@Ni foam electrode exhibits a reduction peak at approximately 0.4 V. This peak is attributed to the conversion of NiO to Ni, as described by eqn (2), as well as the decomposition of the electrolyte, leading to the formation of a solid electrolyte interface (SEI) film.^27^ In subsequent cycles, the peak shifts to around 1.2 V, corresponding only to the conversion process.^28^ The anodic scan of the NiO@Ni foam electrode reveals an oxidation peak at 2.2 V, which corresponds to the reversible formation of NiO, as detailed in eqn (3) (ref. 29 and 30).

2 NiO + 2Li^+^ + 2e^−^ → Ni + Li_2_O

3 Ni + Li_2_O → NiO + 2Li^+^ + 2e^−^

The CV curve of the C@NiO@Ni foam electrode differs from the NiO@Ni foam due to the presence of carbon, which also contributes to lithium-ion storage^6,27^ The electrochemical storage of lithium ions in the electrode can be represented by the following additional chemical reaction eqn (4) and (5):

4 C_6_ + Li^+^ + e^−^ → LiC_6_

5 LiC_6_ → C_6_ + Li^+^ + e^−^

The extra cathodic peaks observed between 0.01 and 0.1 V in the C@NiO@Ni foam electrode are ascribed to the insertion of lithium ions into a graphitic-like carbon structure. The related anodic peaks in the 0.1–1.5 V range are linked to the deintercalation of lithium ions from the carbon structure and the extraction of Li-ions from the pores and defective sites of the electrodes.^31^ The two anodic peaks of C@NiO@Ni foam are also observed at 1.56 V and 2.2 V, corresponding to the partial decomposition of the SEI and the recovery of NiO during delithiation, respectively see eqn (2) and (3). The peak at 1.56 V disappears after the second cycle, indicating the formation of a stable SEI layer. The broadened cathodic peak is supposed a result of the overlapping cathodic peaks of carbon and NiO eqn (2) and (4).

To examine the effect of carbon coating on electrochemical performance, various polymer layers were applied to obtain different amounts of carbon on the surface of NiO@Ni foam electrodes. The comparison is illustrated in ESI(Fig. S1†). The specific capacities at 1C after 100 cycles were 334 mA h g^−1^, 387 mA h g^−1^, and 350 mA h g^−1^ for C@NiO@Ni foam/2, C@NiO@Ni foam/4, and C@NiO@Ni foam/6, respectively. The highest capacity was obtained for C@NiO@Ni foam/4 anode, suggesting that the optimum carbon coating is achieved with four layers of polymers. This provides an ideal balance between sufficient carbon content for conductivity enhancement and structural integrity for electrochemical stability. With additional polymer layers, the coating may become too thick, limiting ion transport and reducing electrochemical performance. Conversely, fewer layers result in inadequate carbon coverage, diminishing the benefits of the conductive coating. A further capacity comparison with NiO@Ni foam was performed using C@NiO@Ni foam/4.

The charge–discharge curves at current density of 0.1C, as illustrated in Fig. 5c and d, are consistent with the CV observations discussed earlier. It is seen that the initial discharge capacities of both electrodes are higher than those in subsequent cycles. This phenomenon can be explained by forming a solid electrolyte interphase (SEI) and the associated redox reactions^32,33^ The initial charge and discharge capacities of C@NiO@Ni foam and NiO@Ni foam are 705/1925 mA h g^−1^ and 604/885 mA h g^−1^, respectively. The initial coulombic efficiency (ICE) was 37% and 68% for C@NiO@Ni foam and NiO@Ni foam, respectively. The lower coulombic efficiency of C@NiO@Ni foam can be attributed to the formation of a porous carbon layer on the surface of the NiO@Ni foam. This layer, being electrochemically active and more porous than the NiO@Ni foam itself, results in increased lithium consumption for the formation of the solid electrolyte interphase (SEI) layer, thereby reducing the coulombic efficiency.^34,35^ Following the first cycle, the capacity stabilized, and consistent, reversible cycling behavior was observed. At the 100th cycle, NiO@Ni foam and C@NiO@Ni foam demonstrated charge/discharge capacities of 678/680 mA h g^−1^ and 475/482 mA h g^−1^, respectively (Fig. 5c). The coulombic efficiency significantly improved by this cycle, reaching approximately 99.6% and 98.5%, respectively.

**Fig. 6:**
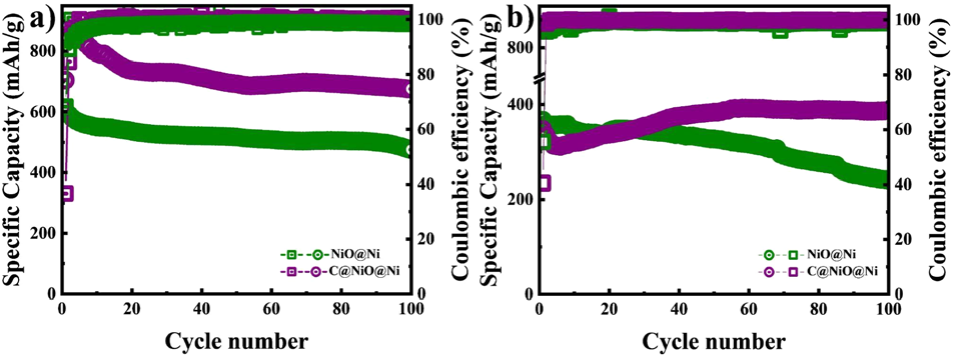
Cycle performance of NiO@Ni foam and C@NiO@Ni foam at a current density of (a) 0.1C and (b) 1C.

Further, a cycle stability test was performed at a potential range of 0.01–3 V at a current density of 0.1C and 1C as illustrated in Fig. 6. The specific capacity of C@NiO@Ni foam was higher than that of NiO@Ni foam at both 0.1C and 1C rates. Specifically, the C@NiO@Ni foam achieved a specific capacity of 678 mA h g^−1^ at 0.1C and 387 mA h g^−1^ at 1C after 100 cycles, compared to the NiO@Ni foam, which showed capacities of 475 mA h g^−1^ at 0.1C and 240 mA h g^−1^ at 1C under the same conditions. It can be seen that carbon-coated and uncoated NiO on Ni foam exhibit slow capacity fading at low current densities. However, their behavior differs at higher capacities: C@NiO@Ni foam shows an initial drop in capacity up to the 6th cycle, followed by an increase, whereas NiO@Ni foam demonstrates continuous capacity fading over 100 cycles. The observed abnormal capacity increase for C@NiO@Ni foam, known as the negative capacity fading phenomenon, can result from three main factors. First, the utilization of the conversion reaction is enhanced due to a lower oxidation state. Second, this improvement is also influenced by the development of a surface layer derived from the electrolyte, which increases the interaction area between the electrolyte and the active materials. Finally, it results from morphological changes in the active materials induced by the rapid lithiation process, as well as the creation of spaces at the interface for lithium storage.^36–38^ Similar patterns are commonly observed in various nanostructured metal oxide electrodes.^39,40^ A comparable phenomenon was observed in NiO anodes by T. P. Nguyen and colleagues.^36^ They demonstrated that NiO anodes did not exhibit negative fading at low current densities, but this phenomenon occurred at higher current densities.^36^

The reversible capacity of C@NiO@Ni foam is superior to that of recently reported NiO@Ni foam, demonstrating its effectiveness in mitigating the volume expansion of NiO and thereby enhancing the electrochemical performance of NiO. The findings indicated superior values in comparison to earlier studies on different NiO composite anodes used for lithium-ion storage, as presented in Table 1.

**Table 1:** Comparison of the electrochemical performance of the current work with previously reported NiO-based composite anodes

Material	Synthesis method	Electrode/mass loading	Current density	Cycle performance	Ref.
C@NiO@Ni foam	CVD and thermal oxidation	Free-standing/4.54 mg cm^−2^	0.5 C	384 mA h g^−1^ after 102 cycles	41
NiO@Ni foam	Thermal oxidation	Free-standing/5.6 mg	∼0.1 C	550 mA h g^−1^ after 50 cycles	17
Biochar-CNT-NiO	Carbonization, MCVD, thermal oxidation	Coated on Cu foil/N/A	0.1 A g^−1^	674.6 mA h g^−1^ after 100 cycles	21
Ni@NiO	Calcination	Coated on Cu foil/0.53–0.88 mg cm^−2^	0.2 A g^−1^	633.7 mA h g^−1^ after 100 cycles	40
Ni/NiO@carbon matrix	Pyrolysis	Coated on Cu foil/N/A	0.2 C	330 mA h g^−1^ after 200 cycles	42
C@NiO@Ni foam	Thermal oxidation with further carbonization	Free-standing/5–6 mg cm^−2^	0.1 C/1 C	678 mA h g^−1^/387 mA h g^−1^ after 100 cycles	This work

EIS was conducted to evaluate the cell resistance using the prepared electrodes after the 5th cycle (Fig. S2†). The Nyquist plots for both NiO@Ni foam and C@NiO@Ni foam electrodes exhibit similar characteristics, featuring a prolonged semicircle in the medium-frequency range, which is commonly associated with the combined resistance of the SEI layer (*R*_f_) and charge transfer (*R*_ct_), and an inclined line in the low-frequency region, representing Li^+^ diffusion within the electrode bulk. The resistance values obtained from the analysis are presented in Table S1.† Notably, the *R*_ct_ of the C@NiO@Ni foam cell is approximately 4 Ohms lower than that of the NiO@Ni foam cell, which can be attributed to the enhanced electrical conductivity provided by the carbon coating.

**Fig. 7:**
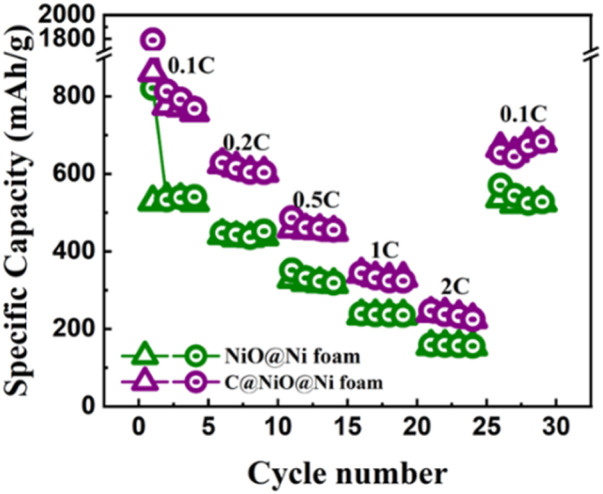
The rate capability of NiO@Ni foam and C@NiO@Ni foam.

As shown in Fig. 7, the discharge capacities of C@NiO@Ni foam at current densities of 0.1, 0.2, 0.5, 1, and 2C were 758, 602, 450, 331, and 225, respectively. Notably, when the current rate is reduced back to 0.1C, the reversible discharge capacity quickly recovers to 680 mA h g^−1^. A similar trend with lower specific capacities of 526, 439, 314, 233, and 154 mA h g^−1^ at the respective current densities is observed for NiO@Ni foam. When returned to 0.1C, it showed a specific capacity of 526 mA h g^−1^. The reduced capacity in NiO@Ni foam can be attributed to the volume expansion of NiO, which causes the NiO nanoparticles to detach from the nickel foam, leading to a loss of electrochemical activity. In contrast, the carbon layer in C@NiO@Ni foam can effectively suppresses this volume expansion, maintaining a higher specific capacity and enhancing the overall battery performance.

**Fig. 8:**
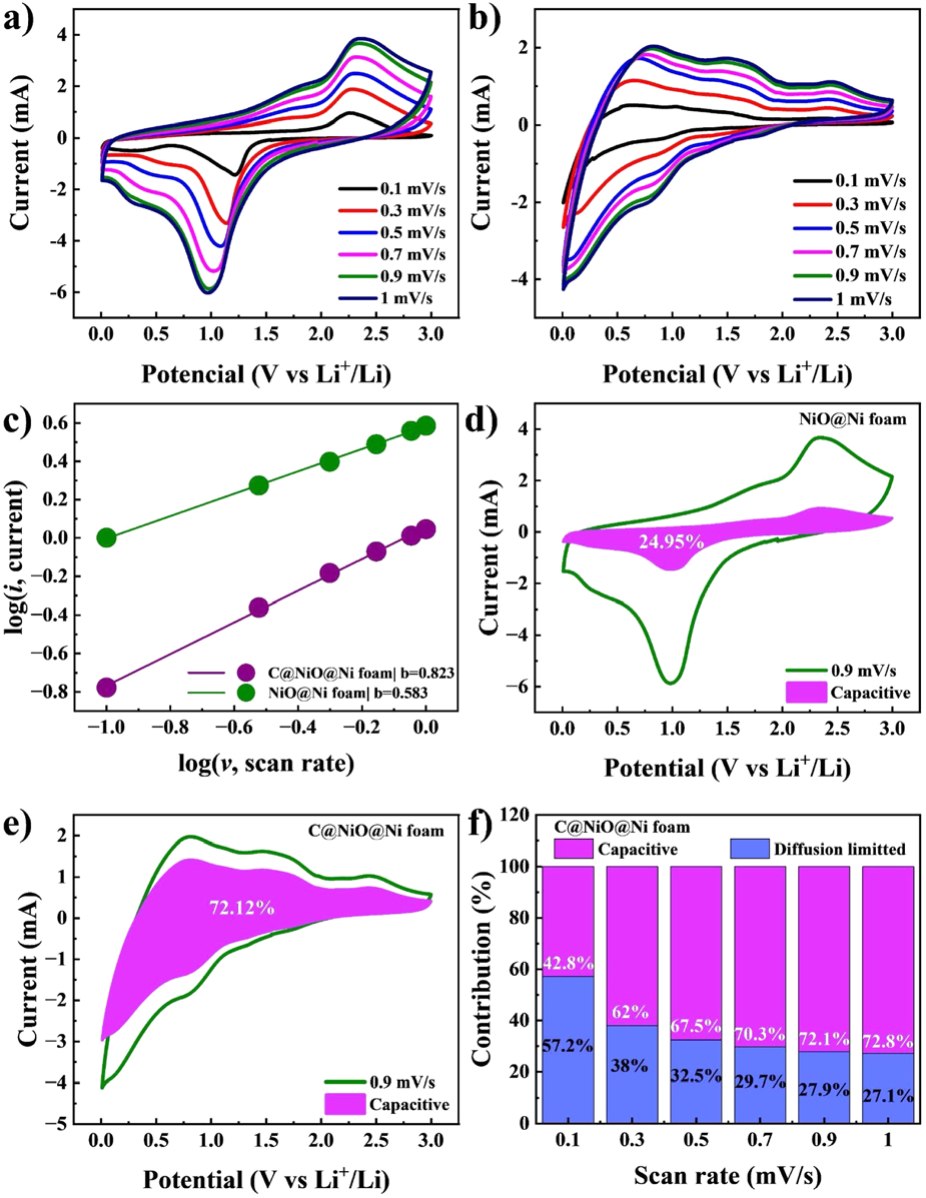
CV curves of (a) NiO@Ni foam and (b) C@NiO@Ni foam at various scan rates (0.1–1 mV s^−1^). (c) A logarithmic linear relationship between current and scan rate. Capacitive storage mechanism contribution at a scan rate of 0.9 mV s^−1^ for (d) NiO@Ni foam and (e) C@NiO@Ni foam. (f) Capacitive storage contribution at various scan rates for C@NiO@Ni foam.

A kinetic study of the NiO@Ni foam and C@NiO@Ni foam anodes was conducted using CV curves at various scan rates to better understand the factors contributing to superior electrochemical performance. Fig. 8a and b illustrates the CV curves at sweep rates ranging from 0.1 to 1.0 mV s^−1^. As established in previous studies, the Li-ion storage mechanism combines capacitive and diffusion-controlled processes, which can be quantitatively analyzed using the following eqn (6) and (7):

6 *i* = *av*^b^

7 log(*i*) = log(*a*) + *b* log(*v*)

where *i* represents the current, *v* denotes the scan rate, and *a* and *b* are adjustable parameters. A value of *b* equal to 1 indicates that a capacitive process dominates the Li-ion storage entirely, whereas *b* equal to 0.5 suggests that a diffusion-controlled process fully governs the storage.

The calculation results are illustrated in Fig. 8c, where the *b* value for C@NiO@Ni foam reaches 0.823, significantly higher than the 0.583 value for NiO@Ni foam. This suggests that C@NiO@Ni foam exhibits more optimized capacitive kinetics. The ratio of surface- and diffusion-controlled charge storage mechanisms can be further calculated at a specific voltage using eqn (8):

8 *i*(*V*) = *k*_1_*ν* + *k*_2_*ν*^1/2^

The capacitive contribution of C@NiO@Ni foam significantly rises from 42.7% to 72.8%, when the scan rate increases from 0.1 to 1 mV s^−1^, as illustrated in Fig. 8f. Specifically, at a scan rate of 0.9 mV s^−1^, the shaded area showing the capacitive charge for C@NiO@Ni foam reaches 72.1%, whereas it is only 24.9% for NiO@Ni foam. These results indicate that the carbon-coated NiO nanostructure greatly enhances lithiation kinetics, leading to better reversibility at higher rates.

## Conclusion

In summary, a carbon-coated NiO anode was successfully fabricated on Ni foam and demonstrated as a 3D material for lithium-ion batteries (LIBs). Characterization techniques, including XRD, SEM, and EDS, confirmed the formation of the carbon-coated NiO on the Ni foam substrate. The unique 3D architecture, with its extensive surface area, promoted efficient electrode–electrolyte contact and reduced Li-ion diffusion pathways. Additionally, the carbon coating not only preserved structural integrity during cycling but also improved the electronic conductivity of NiO. Consequently, the carbon-coated NiO anode exhibited significantly enhanced performance, delivering a capacity of approximately 678 mA h g^−1^ at 0.1C and 387 mA h g^−1^ at 1C after 100 cycles. In contrast, the uncoated NiO anode showed capacities of 475 mA h g^−1^ at 0.1C and 240 mA h g^−1^ at 1C under the same conditions. This notable improvement underscores the potential of carbon-coated NiO foam as a high-performance and reliable anode material for advanced LIB applications.

## Data availability

The data supporting this article have been included as part of the ESI.†

## Author contributions

Nurbolat Issatayev: writing—original draft preparation; visualization. Diana Abdumutaliyeva: experiments; conceptualization; data curation. Yerbolat Tashenov: visualization. Dossym Yeskozha: experiments, validation; investigation; data curation. Adilkhan Seipiyev: experiments. Zhumabay Bakenov: analysis, resources, proofreading and editing. Arailym Nurpeissova: conceptualization; supervision; resources; funding acquisition; analysis; writing—review and editing.

## Conflicts of interest

The authors declare no conflicts of interest.

## Supplementary Material

RA-014-D4RA07119K-s001

RA-014-D4RA07119K-s002

RA-014-D4RA07119K-s003

RA-014-D4RA07119K-s004
