# Supplementary figures and images for: Three-dimensional carbon coated and high mass-loaded NiO@Ni foam anode with high specific capacity for lithium ion batteries
Source: RSC Adv. 2024 Dec 23;14(54):40069–76. doi: 10.1039/d4ra07119k (PMC11664242; doi:10.1039/d4ra07119k)

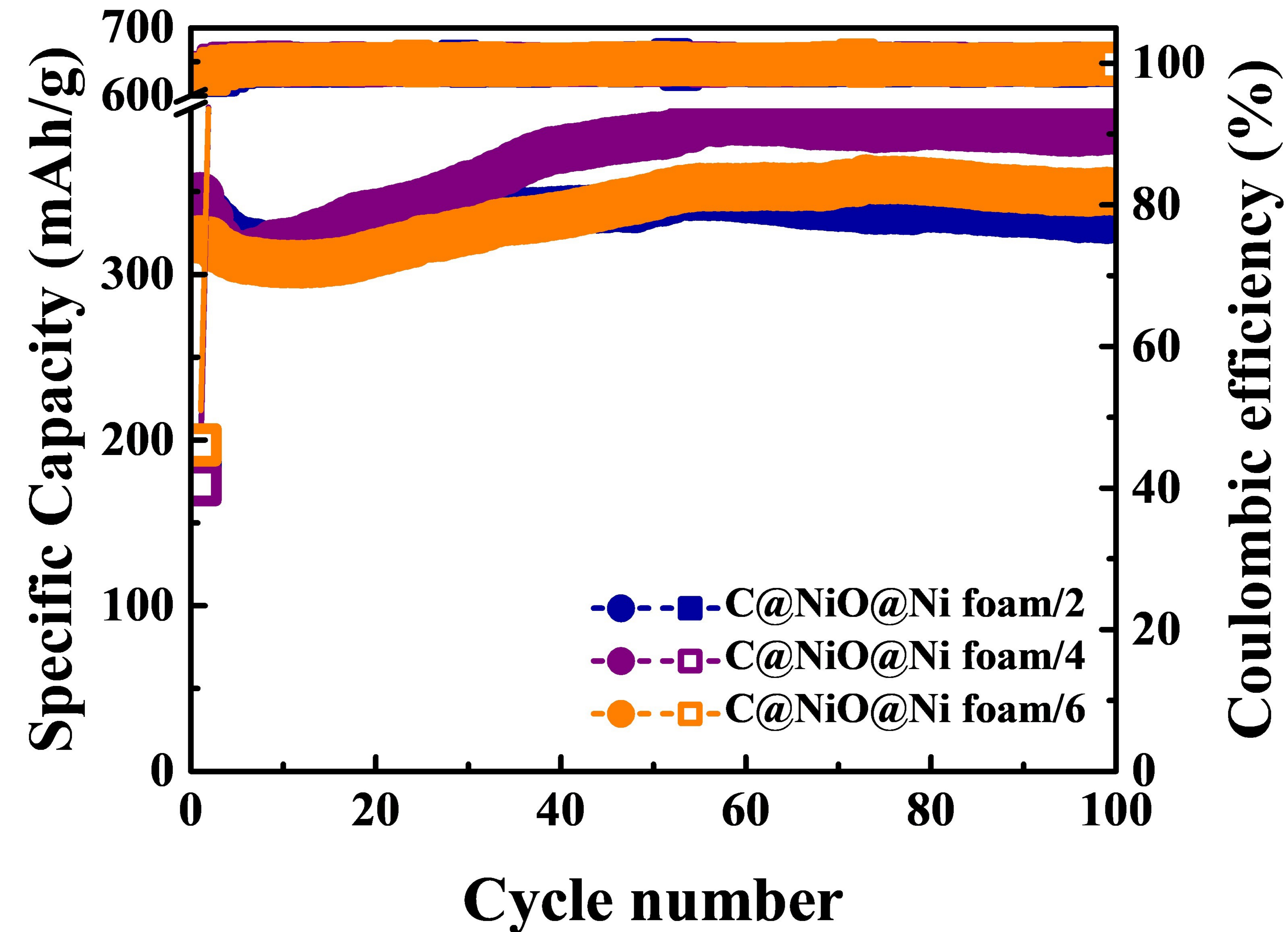

Supplement: RA-014-D4RA07119K-s002 [file RA-014-D4RA07119K-s002.pdf]

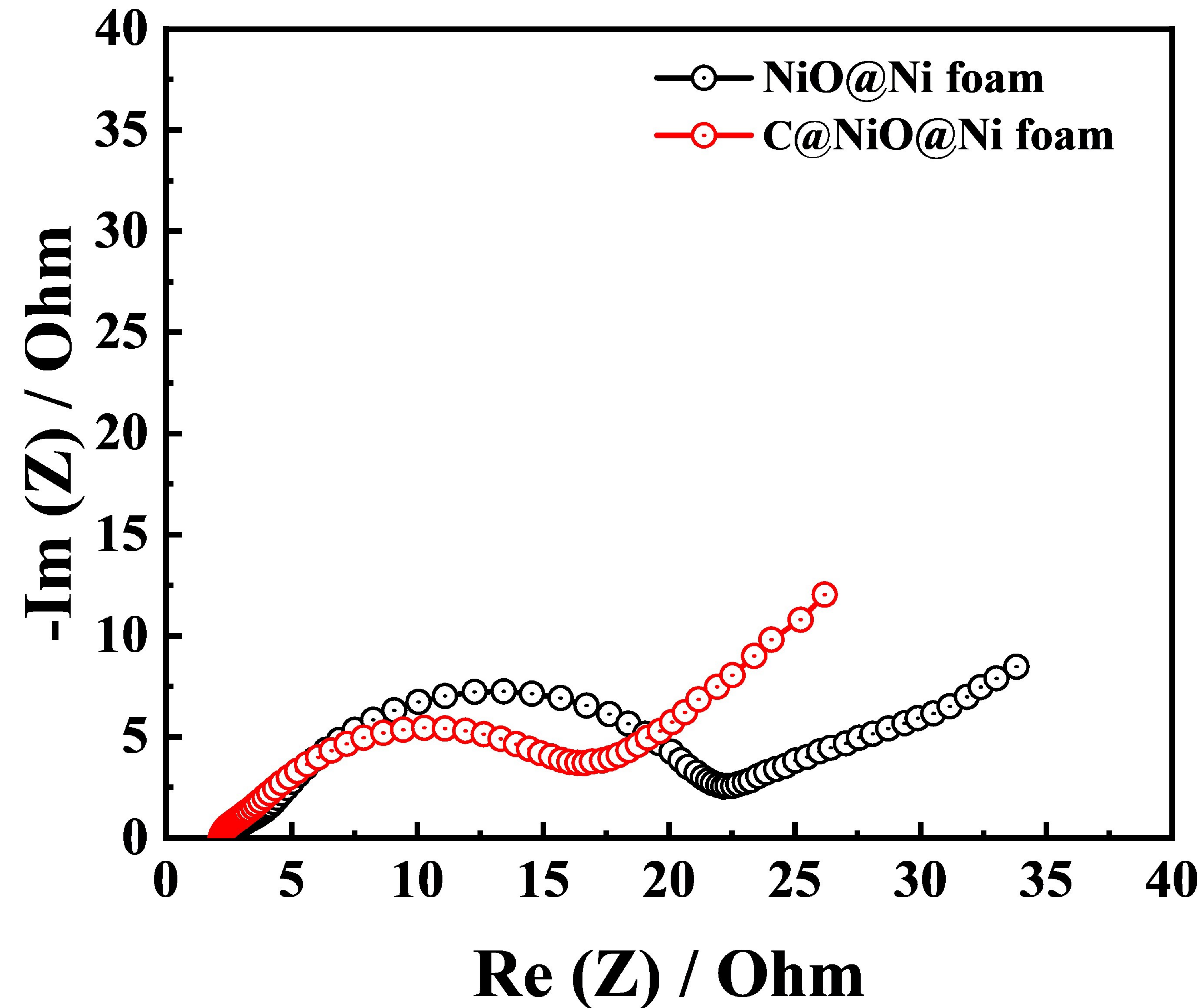

Supplement: RA-014-D4RA07119K-s003 [file RA-014-D4RA07119K-s003.pdf]
