# Supplementary material for: Three-dimensional carbon coated and high mass-loaded NiO@Ni foam anode with high specific capacity for lithium ion batteries
Source: RSC Adv. 2024 Dec 23;14(54):40069–76. doi: 10.1039/d4ra07119k (PMC11664242; doi:10.1039/d4ra07119k)
Supplement: RA-014-D4RA07119K-s004 [file RA-014-D4RA07119K-s004.pdf]

Supplementary Information (SI) for RSC Advances.
This journal is © The Royal Society of Chemistry 2024

Supporting Information

Three-Dimensional Carbon Coated and High Mass-Loaded NiO@Ni foam Anode with High Specific Capacity for Lithium Ion Battery

Nurbolat Issatayev,[a] Diana Abdumutaliyeva,[b] Yerbolat Tashenov,[b] Dossym Yeskozha,[a] Adilkhan Seipiyev,[a] Zhumabay Bakenov*[a, c, d] and Arailym Nurpeissova*[a, c]

[a.] Institute of Batteries, 53 Kabanbay Batyr Ave., Astana 010000, Kazakhstan

[b.] Department of Chemistry, L.N. Gumilyov Eurasian National University, 2 Satpayev St., Astana, 010008 Kazakhstan

[c.] National Laboratory Astana, Nazarbayev University, 53 Kabanbay Batyr Ave., Astana, 010000, Kazakhstan

[d.] Department of Chemical and Materials Engineering, Nazarbayev University, 53 Kabanbay Batyr Ave., Astana, 010000, Kazakhstan

** Corresponding Author: arailym.nurpeissova@nu.edu.kz; zbakenov@nu.edu.kz*

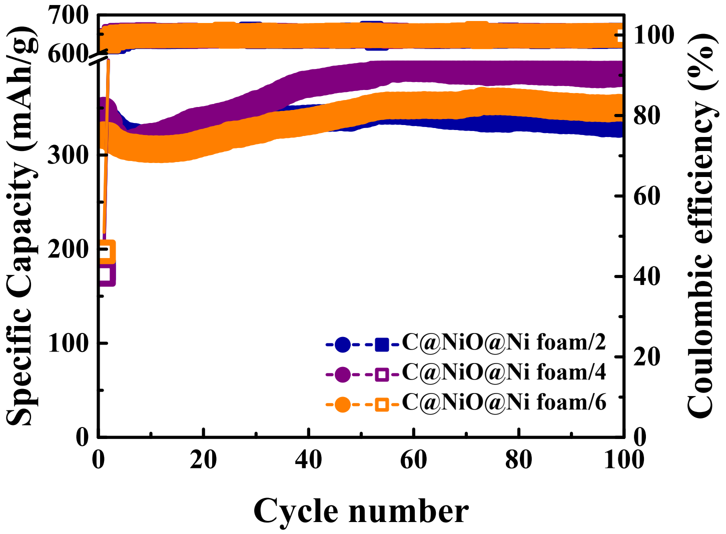

Figure S1. Cycle performance of C@NiO@Ni foam/2, C@NiO@Ni foam/4, and C@NiO@Ni foam/6 at a current density of 1C.

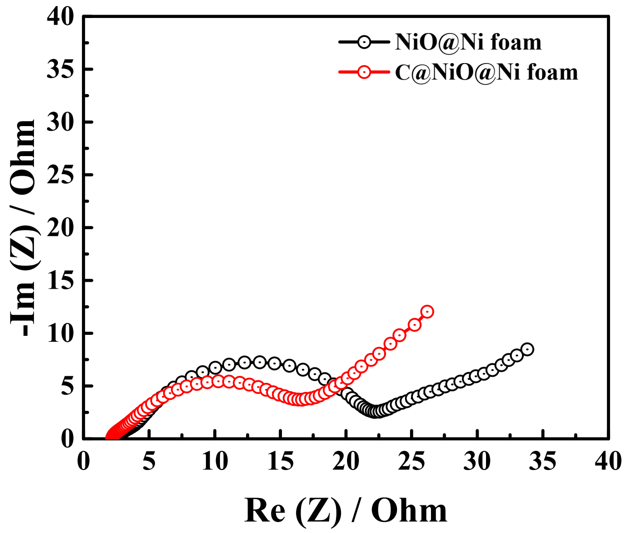

Figure S2. Nyquist plots of the NiO@Ni foam and C@NiO@Ni foam measured after 5th cycle.

Table S1. Fitting results of the Nyquist plots of the NiO@Ni foam and C@NiO@Ni foam.

	5th cycle		
	R_s, Ω	R_f, Ω	R_{ct}, Ω
NiO@Ni foam	2.74	4.23	14.98
C@NiO@Ni foam	2.16	4.09	11.12
